# The individual, place, and wellbeing – a network analysis

**DOI:** 10.1186/s12889-021-11553-7

**Published:** 2021-09-06

**Authors:** Eoin McElroy, Mathew Ashton, Anne Marie Bagnall, Terence Comerford, Mick McKeown, Praveetha Patalay, Andy Pennington, Jane South, Tim Wilson, Rhiannon Corcoran

**Affiliations:** 1 Department of Neuroscience, Psychology and Behaviour, University of Leicester, Leicester, UK; 2 Department of Public Health and Policy, University of Liverpool, & Liverpool City Council, Liverpool, UK; 3 Centre for Health Promotion Research, Leeds Beckett University, Leeds, UK; 4 NIHR ARC North West Coast, Liverpool, UK; 5 School of Nursing, University of Central Lancashire, Preston, UK; 6 Centre for Longitudinal Studies and Medical Research Council Unit for Lifelong Health and Ageing, University College London, London, UK; 7 Institute of Population Health Sciences, University of Liverpool, Liverpool, UK; 8 Department of Primary Care and Mental Health, University of Liverpool, Liverpool, UK

**Keywords:** Network analysis, Wellbeing, Place, Neighbourhoods

## Abstract

**Background:**

Previous research has examined individual-level and place characteristics as correlates of subjective wellbeing, with many studies concluding that individual factors (e.g. health, finances) are more strongly related to wellbeing. However, this ‘dualistic’ approach has been challenged, with some arguing that it is impossible to disentangle the effects of the two domains, and that wellbeing should be considered as part of a network of mutually reinforcing relationships between individual, community and place characteristics. We used network analysis to explore these complex associations.

**Methods:**

Data were from a large sample of adults from a socioeconomically disadvantaged region of the United Kingdom (*N* = 4319). Wellbeing was assessed using the 7-item version of the Warwick–Edinburgh Mental Wellbeing Scale (SWEMWBS). Mixed graphical networks were estimated including wellbeing, place and individual-characteristic variables as nodes.

**Results:**

We found a densely connected network in which wellbeing was associated, both directly and indirectly, with all of the individual, community and place characteristics assessed. Wellbeing was most strongly connected with individual characteristics, in particular financial difficulty and subjective physical health. However, controlling for all other variables in the network model, wellbeing was positively associated with local greenspace usage, civic agency, and neighbourhood cohesion, and negatively associated with housing disrepair. Greater specificity in these associations was observed when the wellbeing construct was broken down into its constituent parts.

**Conclusions:**

These findings highlight the complex relationships that exist between individual, community and place characteristics in the context of subjective wellbeing, and that all domains need to be considered when developing population-level strategies to improve wellbeing. Further consideration needs to be given to how this might happen in practice, for example through a combination of consistent use of community engagement methodologies alongside Health in All Policy (HiAP) approaches.

**Supplementary Information:**

The online version contains supplementary material available at 10.1186/s12889-021-11553-7.

## Background

Despite continuing controversy over issues of definition and measurement as well as terminology, it is clear that wellbeing has risen up the list of public health concerns [1, 2]. Most wellbeing research focusses on personal, subjective assessments of one’s feelings (i.e. eudaimonic and hedonic wellbeing) or assessments of variables relative to day-to-day living (e.g. local economy and safety). However researchers are increasingly broadening the concept of wellbeing beyond the level of the individual to encompass aspects of the community (e.g. shared values, belonging and ownership of community processes) [3]. Personal-subjective wellbeing, along with community wellbeing [3], are increasingly acknowledged as important population outcomes with advances made in their representation within international policy agendas [4–6]. In part this is because, as with health, there exist not only individual level determinants or correlates of wellbeing but also significant wider economic, environmental and social determinants. These wider determinants reflect systemic inequities in the living circumstances of individuals and communities that are unjust and avoidable and can often arise as unwanted consequences of well-meaning but short - sighted or poorly targeted policy interventions [7, 8]. As a result, there have been heightened calls for wellbeing in all policy approaches, as enacted in the Wellbeing of Future Generation Act 2015 in Wales [6]. Research conducted under the auspices of the Community Wellbeing Evidence Programme of the What Works Centre for Wellbeing has been part of a broader effort that has begun to identify the characteristics of UK places that may contribute to poor wellbeing or languishing or that may be related to local disparities in personal wellbeing (i.e. wellbeing inequalities) [9, 10].

Although some authors argue that individual characteristics (e.g. personal health, financial status) are more robustly associated with subjective wellbeing than community or place characteristics [11], others suggest that individual, community and place characteristics should not be considered independently from one another. This is because they likely operate in a complex system of reciprocal relationships [12] as can be shown in research using mobile data collection methods for example [13]. It is incontrovertible that one’s relative flourishing or languishing will depend, at least in part, on what resources we have available to us in our neighbourhoods and communities. The place-based resources that support high levels of wellbeing are numerous but include high quality employment or other forms of occupation, enjoyable and immersive cultural and activity-based pursuits, access to good quality food and other forms of retail, reliable social support from those around us as well as wider forms of social capital, sense of belonging to and meaningful involvement with your neighbourhood and community, access to good quality open/green spaces, housing and neighbourhood living environment [14–23].

Subjective and community wellbeing, individual characteristics, and the living environment: a complex network approach.

Further research into the complex associations between subjective and community wellbeing, place, and individual factors is warranted. The network approach [24, 25] is a promising conceptual and statistical framework for such inquiries. This approach conceptualises psychological phenomena as complex systems, wherein aetiological factors and psychological indicators (e.g. moods, behaviours) influence one another directly in a cycle of mutual reinforcement [26]. Network studies have seen notable growth in the field of mental health research in recent years. Such models are presented graphically; variables take the form of nodes (points in space), with lines linking nodes (referred to as edges) denoting the presence, strength and direction of associations between variables. In most psychological networks, edges reflect conditional dependencies; i.e. the association between variables controlling for all other variables in the network [27]. The overall connectivity of each node can be quantified using a series of metrics known as centrality, which allow us to identify the nodes that are most important to the network as a whole. Furthermore, by focusing on direct and indirect associations, network models offer detailed and nuanced insights into the associations that connect different domains of variables. Thus, network models may help clarify the complex relationships and pathways that connect individual, place-based and community characteristics with elements of hedonic and eudaimonic wellbeing. This in turn may broaden our understanding of wellbeing beyond simple eudemonic/hedonic experiences, and help establish the idea that wellbeing is a complex system consisting of individual and community elements.

To our knowledge, only one study so far has used network analysis to investigate subjective wellbeing [28]. This study focussed entirely on the associations between different aspects of hedonic and eudaimonic wellbeing, which were assessed using individual items from the Warwick-Edinburgh Mental Well-being Scale (WEMWBS). They found that items related to positive self-perception and mood were most central across four large general population samples [28]. To date, no studies have used psychological network analysis to investigate associations between subjective wellbeing and its wider determinants or broader indicators of wellbeing (e.g. community wellbeing). One study [29] has used psychological network analysis to explore how relative disadvantage and the neighbourhood environment are related to self-reported mental distress collected using continuum measures of depression, anxiety and feelings of persecution. This study, confirmed that connections across the different variable domains of neighbourhood environment and mental distress altered with level of neighbourhood deprivation, illustrating and demonstrating the important negative psychological consequences of living in more disadvantaged neighbourhoods.

In the current exploratory study, we used network analysis to examine the associations between psychological wellbeing and both individual and place characteristics. As network analysis is a highly flexible approach, allowing for investigations at different levels of granularity [30], we examined two distinct network structures: i) a network in which psychological wellbeing was treated as a uni-dimensional construct (i.e. the composite score on the short WEMWBS [31]), and ii) a network in which wellbeing was broken down into its constituent parts (i.e. the individual items from the short WEMWBS). The overall aim of this research was to identify the individual and living environment characteristics that are most strongly associated with overall subjective wellbeing, as well as those associated with the individual wellbeing items that comprise the short WEMWBS.

## Methods

### Participants

During the latter half of 2015 and the beginning of 2016, data were collected from residents of neighbourhoods in the North West Coast of England as part of the National Institute of Health Research Collaboration for Leadership in Applied Health Research and Care North West Coast (NIHR CLAHRC NWC) Household Heath Survey. A total of 4319 people were surveyed from within a sample that was drawn from an area of high national deprivation. Within this economically disadvantaged population, a random probability sample was taken from 20 high-deprivation areas, and 8 relatively low-deprivation areas. Each area had a population of approximately 10,000 people and the majority of areas were defined by electoral ward boundaries. The areas were selected based on the following considerations: population size (5000–10,000 people), level of disadvantage (as measured via Index of Multiple Deprivation), coherent shared identity, and infrastructure for policy delivery. One adult participant was surveyed per household. Fifty-seven per cent of the sample identified as female, and ages ranged from 18 to 95 (M = 49.12, SD = 19.13). The majority of the sample (89%) reported having white ethnic backgrounds. Further details of the demographic characteristics of the sample are provided in the online supplement (Table S1).

### Measures

#### Wellbeing

Wellbeing was assessed using the 7-item version of the Warwick–Edinburgh Mental Wellbeing Scale (SWEMWBS, see methods S2 for licencing information) [31]. The SWEMWBS is a short-form version of the original WEMWBS [32], which was designed to capture population mental wellbeing, acknowledging that mental health is more than the absence of mental illness [31]. Items tap both hedonic and eudemonic aspects of wellbeing, with responses indicated on a 5-point Likert scale ranging from ‘1 = None of the time’ to ‘5 = All of the time’. The SWEMWBS has demonstrated good psychometric properties in UK general population samples [33]. In order to examine the associations between wellbeing and place characteristics at different levels of granularity, two networks were estimated in the present study: one in which wellbeing was treated as a composite variable (component scores from a uni-dimensional principal components analysis) and one in which the 7 items of the SWEMWBS were entered as unique nodes within the network.

Additional variables in our network analysis can be grouped into two broad domains; place characteristics (factors related to housing and the local neighbourhood/community) and individual characteristics (e.g. socioeconomic status, self-reported physical health). Our data were a mix of continuous, binary categorical and count variables. Full details of the specific variables included (i.e., exact survey questions, original scale, data manipulation and construction of composite measures) are available in Table S1, but are also summarised below.

### Place-based and living environment factors

We included measures of:

i) Household crowding. This was computed as the ratio of bedrooms to number of residents in the household.
ii) Housing quality. This was a composite variable including questions related to having sufficient heating, the presence of mould and of condensation during cold times of the year.
iii) Neighbourhood cohesion/social capital. This composite variable was based on questions about trust, relationships and sense of belonging to the neighbourhood.
iv) Neighbourhood disorder. This composite variable included questions pertaining to neighbourhood problems of vandalism, troublesome neighbours and sense of discrimination.
v) Greenspace usage. This composite variable was made up of questions about the usage of neighbouring parks, open spaces and recreation areas.
vi) Club/organisation involvement. This variable was made up of a count of club/organisation membership.
vii) Civic agency. This was measured with one question asking whether individuals felt they could influence decisions made about their neighbourhood.

All composite scores were component scores derived from uni-dimensional principal components analyses (PCAs). Composite scores were utilised in place of individual items for two reasons. First, we were mainly interested in overall phenomena (e.g. greenspace usage) based on causal indicators (e.g. usage of parks, allotments etc.), thus the measurement models could be considered formative [34]. Second, from a statistical point of view, we were keen to avoid topological overlap by including overly-similar items, as this may inflate the importance of certain variables within the network [26]. Component scores were calculated and saved using SPSS v25 [35]. A detailed breakdown of which variables were used to derive the composites (and accompanying component loadings) is provided in Table S1.

#### Individual factors

Included:

i) Employment status which was coded simply as gainfully employed or not.
ii) Marital status which categorised participants as either single or as married, co-habiting or in civil partnership.
iii) Education which was recorded as at degree level or less.
iv) Home ownership that included categories of owning the home whether outright or with mortgage or renting/other.
v) Non-paid caring responsibilities coded as either none, or as caring for another person for 1 or more hours per week.
vi) Financial difficulty was coded as either doing well/ getting by, or struggling financially.
vii) Religiosity was based on Likert scale responses to the question “To what extent do you agree or disagree that your personal religious beliefs or faith are important to you?”
viii) Social support/friendships. This composite variable included questions related to frequency of contact with friends and access to social support.
ix) Subjective physical health. For this variable respondents were asked to self-report how physically healthy they feel on a 100 point scale with higher values reflecting greater health.

#### Additional demographic covariates

The following demographic covariates were included in the estimation of the networks, but were not visualised in subsequent network graphs.

i) Gender (0 = male; 1 = female)
ii) Age in years
iii) Ethnicity (0 = White British/Irish; 1 = else)
iv) Sexual orientation (0 = heterosexual; 1 = else)

### Analysis

Missing data were generally low, with less than 1% of scores missing on 13 of our 21 variables. Age had the highest proportion of missing values at 11%. Missing data were imputed using the R package missForest [36]. This package uses an iterative imputation method based on random forests [36]. This non-parametric approach is particularly effective at imputing mixed-type data [36, 37].

The majority of psychological networks to-date have been modelled as Pairwise Markov Random Field (PMRF), a broad class of statistical model [38]. PMRFs consist of nodes (elements represented as points in space) and edges (lines connecting nodes indicating conditional dependence relations). The type of PMRF used in psychological networks depends on the nature of the data. Most studies in this field have used methods that were developed specifically for continuous (Gaussian graphical models [39]) or binary data (Ising model [40]). Given our data comprised a combination of continuous, binary categorical and count data, mixed graphical networks were estimated using the ‘mgm’ package [41], which was developed to estimate networks using mixed-type data. The mgm procedure combines mixed joint distributions with a structure estimation approach based on generalized covariance matrices [42]. This produces a network structure in which all variables are modelled in their correct domain [42]. Moreover, such networks can be interpreted in the same way as the more well-known Gaussian and binary networks; variables are presented as nodes (points in space) and the associations between these variables are presented as edges (lines connecting variables, with colour and thickness denoting direction and strength of associations). Edges in the network can be thought of as conditional dependencies, i.e. they represent the association between two variables controlling for all other variables in the network. In a practical sense, edges can be interpreted as partial correlation coefficients [27]. In order to reduce the likelihood of spurious edges in the network, *l*1-regularization is applied to shrink edges and set very small edges to 0 [41].

Networks were visualised using the ‘qgraph’ package [39], which employs the Fruchterman-Reingold algorithm [43] to plot strongly associated nodes closer together. Nodes with few and/or weak connections are thus relegated to the periphery of the network. The overall importance of each node within its network (i.e. centrality) was quantified in the form of node strength. Strength is calculated by summing the absolute values of the edge weights of a given node, and can be thought of as a measure of the direct influence that a node exerts over the larger network [44]. Strength values are presented as standardised Z-scores, with higher values reflecting greater importance within the network. Edge weight accuracy and centrality stability (i.e. the degree of confidence with which edge weight and centrality rankings can be interpreted) were assessed using the ‘bootnet’ package [27]. Descriptions of these processes are available in the online supplementary materials (SMethods 1).

As the aim of this study was to explore the associations between subjective wellbeing, neighbourhood, and individual characteristics, we chose to focus our discussion on these nodes only, and treated demographic variables (age, gender, sexual orientation and ethnicity) as control variables. We therefore estimated the networks including these covariates as nodes, but visualised only the nodes related to wellbeing and place by sub-setting the weighted adjacency matrices. Network visualisations with covariates included as nodes are available in the supplementary materials.

## Results

Descriptive statistics for all variables included in our network analyses are presented in the online supplementary materials (Table S1).

### Overall wellbeing, individual and neighbourhood characteristics

**Fig. 1:**
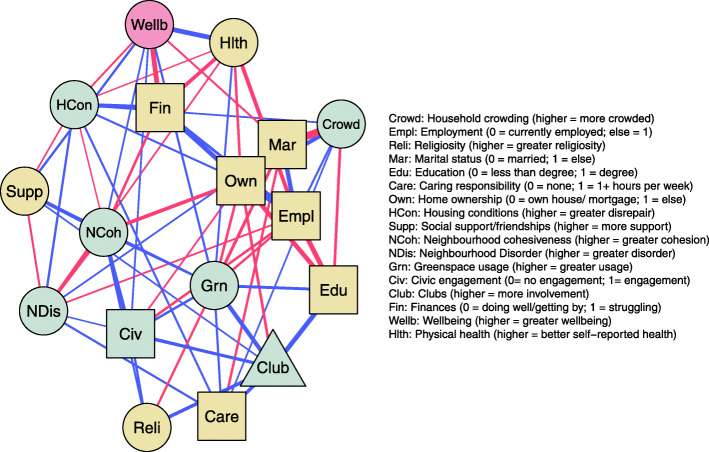
Mixed graphical network of individual and place characteristics and overall wellbeing, controlling for demographic factors. Blue edge = positive association. Red edge = negative association. Circle nodes = continuous variables. Square nodes = binary variables. Triangle nodes = count variables. Grey nodes = place characteristics. Yellow nodes = individual characteristics

**Fig. 2:**
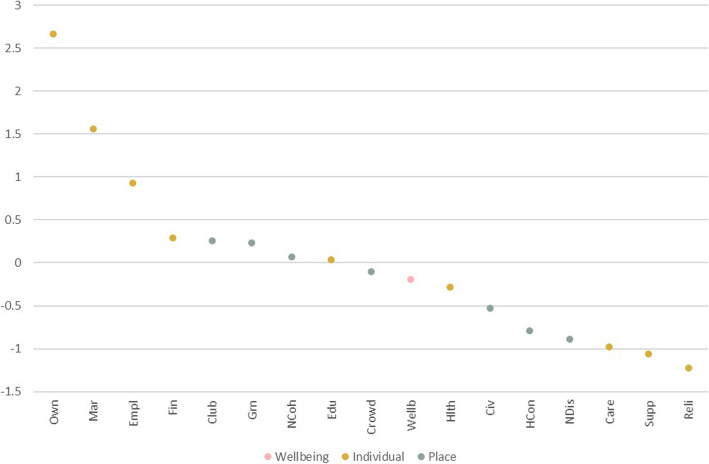
Strength values demonstrating importance of each node within the network. Presented as standardised Z-scores with higher values reflecting greater importance

The overall network contained 98 non-zero edges out of a possible 210 (47% of nodes were directly connected), indicative of the many and complex pathways between wellbeing, individual factors and neighbourhood characteristics. The tests of network accuracy and stability are presented in the online supplementary materials (Fig. S1-S2). Correlation stability coefficients were high (≥0.75) for both edge weights and strength centrality, indicating that the rank ordering of edges and centrality metrics can be interpreted with confidence, and the network can be considered both reliable and accurate. Figure 1 presents the edges between wellbeing, individual and neighbourhood characteristics. A full visualisation of the 21-node network (including demographic covariates) is available in the online supplementary materials (Fig. S3). Strength values for this network are presented in Fig. 2. Home ownership was the most influential node within the network, followed by marital status and employment. Religiosity had the lowest strength.

Non-zero edge weight values (i.e. strengths of connections) for the wellbeing node are presented in Table S2. Wellbeing was most strongly connected with individual characteristics, in particular financial difficulty and subjective health. With regards to place characteristics, controlling for all other variables in the network model, wellbeing was positively associated with local greenspace usage, civic agency, and neighbourhood cohesion, and negatively associated with housing disrepair.

### Wellbeing domains, individual and neighbourhood characteristics

**Fig. 3:**
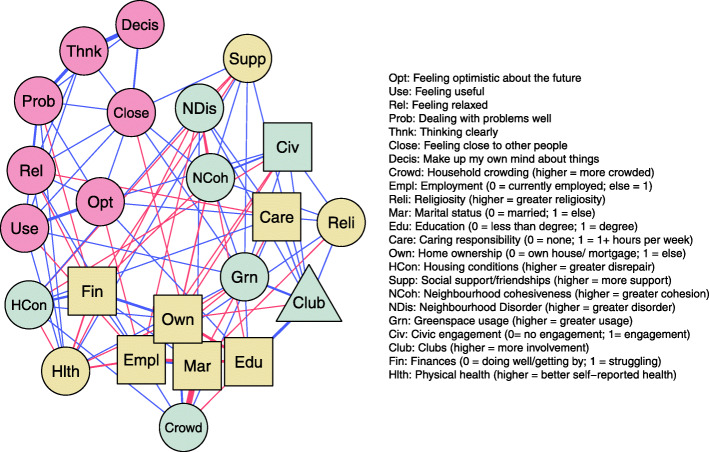
Mixed graphical network of individual and place characteristics and wellbeing features, controlling for demographic factors. Blue edge = positive association. Red edge = negative association. Circle nodes = continuous variables. Square nodes = binary variables. Triangle nodes = count variables. Grey nodes = place characteristics. Yellow nodes = individual characteristics

Our second network, in which all 7 items of the SWEMWBS were included as nodes, is presented in Fig. 3. A total of 132 edges (37%) in this network were non-zero. Non-zero edge weight values for the 7 wellbeing nodes are presented in Table S3. Looking at the individual characteristics, the strongest edge was a negative association between financial difficulty and the item “I’ve been feeling relaxed”. The edge between social support and “I’ve been feeling close to other people” was also notably strong. With regards to neighbourhood characteristics, neighbourhood cohesion was positively associated with the item “I’ve been feeling close to other people”. Civic agency (i.e. participants feeling that they could influence local decisions) was positively associated with the item “I’ve been feeling optimistic about the future”. Greenspace usage was positively associated with both of the above wellbeing items.

## Discussion

This study set out to explore the connections between and amongst individual and neighbourhood, place-based factors that, together, go some way to represent the intricate relationships within a network of variables that contribute to subjective wellbeing as measured by the SWEMWBS. We found that this network of individual, neighbourhood and wellbeing variables was densely connected, highlighting the complex relationships that exist between individual and place characteristics in the context of subjective wellbeing.

Our findings agree with the previous literature such as Propper et al. [11] that argues that individual characteristics are the strongest predictors of subjective wellbeing. In this analysis, subjective financial difficulty and physical health had the strongest connections with overall wellbeing represented in the composite Short WEMWBS score. However, notwithstanding the importance of perceived financial difficulty and overall health, we found that wellbeing is robustly associated with place characteristics, even after controlling for established individual-level correlates. This finding alone supports the conclusion that individual factors and neighbourhood/place-based factors need to be considered together in a relational way if we are to properly understand subjective wellbeing as it is spatially distributed across nations [12] and so that we can effectively intervene to improve wellbeing at national policy levels [7].

The results reported here highlight that areas characterised by lack of accessible open space, civic disengagement, a lack of neighbourhood cohesion, and housing disrepair seem to be at particular risk of low wellbeing. As such our findings are consistent with the reviews led by Marmot [45, 46] in stressing that place matters for our health and wellbeing and that people’s wellbeing is significantly affected by systemic place-based conditions over which they have very little, if any, control. These findings are also consistent with the prior research base as reviewed and reported by the community wellbeing evidence programme of the What Works Centre for Wellbeing [17, 18, 23] and supported by a recent secondary data analysis conducted by Curtis et al. [10] as part of that programme.

Our analysis highlighted that using local open or greenspace was more strongly associated with wellbeing than any other place-based/neighbourhood factor. In the context of the recent restrictions imposed in relation to control of COVID-19, the importance of open space usage is all the more pointed. According to the ONS [47], while 1 in 8 British households has no garden, 28% of people live within 5 min walk of a park. Furthermore, it seems from the ONS analysis that people living in the most deprived communities of England are twice as likely to live within 5 min of a local park or open space than those living in less deprived areas. In the North West of England, where the data analysed in the current research was collected, 30.8% of the population is reported to live within 5 min walk of a local park, rising to 55.5% when playing fields are added in [47]. This recently published information suggests that it is not availability of local open space assets per se that is the issue in relation to wellbeing but rather it is the use of those assets that is the important determinant. Therefore, there needs to be further research exploring the barriers to greenspace usage that should include a consideration of the quality of the walking journey to and from local open spaces in disadvantaged neighbourhoods.

Nevertheless, in highlighting the importance of using open spaces, our finding agrees with the wealth of published multi-disciplinary research, supported via a widely accepted theory [19], that emphasises access to and use of greenspace as being part of the public health solution to address physical health challenges, mild to moderate mental health difficulties and low wellbeing [20–22]. Previously, it has been difficult to judge whether the use of local open space held more promise in this regard than other available intervention options [7]. This network analysis, provides some information in this regard by finding that of the neighbourhood factors measured in this analysis, use of local open space was the one most strongly associated with subjective wellbeing. It seems therefore that the accessibility, stewardship and management of local public spaces merits attention at policy level if the ambition is to improve subjective wellbeing at scale. As Snaith [48] points out, however, different cultures are likely to have different preferences for the type of public spaces they will use. With the sample analysed here being of predominantly white British origin, we must bear in mind the potential consequences of rolling out a uniform approach to the aesthetic design of parks and open spaces.

This research not only used network analysis to explore the factors associated with overall subjective wellbeing as measured by the short WEMWBS, it also embraced the complexity of the concept itself heeding the fact that this composite score is comprised of distinct aspects or different forms of subjective wellbeing. By analysing the data at the level of the individual question, we found that different neighbourhood/ place-based factors were associated with different aspects of wellbeing. In line with the overall importance of use of local greenspace to the composite wellbeing score, we found that this variable was particularly associated with the SWEMWBS questions of ‘feeling close to others’ and ‘feeling useful’. These more specific wellbeing findings relating to use of local greenspace may suggest that these areas are places where neighbours and members of the community may meet or bump into each other enabling a feeling of closeness. That use of these spaces was also associated with feeling useful may relate to the purpose of going to the areas and what activities are pursued when there.

Other notable links between individual SWEMWBS questions and neighbourhood variables included that an increased sense of neighbourhood cohesion was directly connected to the feeling of being close to others, supporting the sense that the connections being detected with the network have face validity. The finding that civic agency seemed to be most directly linked to having a sense of optimism for the future is reminiscent of previous research that emphasises the importance of joint decision-making in communities to wellbeing [18] and also to findings indicating that deciding about neighbourhood outcomes, purposes and visions in the future is an inherently optimistic process [49].

The present study has both strengths and limitations. Strengths include a novel and sophisticated analytical approach, and a dataset with rich information on both individual and place characteristics and mental wellbeing. In terms of limitations, the overall sample, although drawn from neighbourhoods of varying levels of relative deprivation, focussed on an economically deprived area of the United Kingdom, and thus findings may not generalize to areas characterised by greater social/economic advantage or less inequality. Indeed, as socioeconomic position is associated with dis/advantages across both individual and place domains, greater inequality may serve to moderate the relationships observed in the present networks. In addition, as is the case with all cross-sectional network analyses, dynamic associations and causality cannot be established between variables. However cross-sectional networks such as these are useful as an exploratory tool and can be used to identify potential causal pathways without relying on the stringent assumptions (e.g., acyclicity) of other methods (e.g. directed acyclic graphs) [50]. Future research could seek to use intensive longitudinal data and emerging dynamic network methodologies to explore the direction and temporality of theses associations, which would further unpack these complex processes. Although we employed a widely-used, reliable and valid measure of subjective wellbeing, the SWEMWBS is largely based on hedonic and eudaimonic conceptualisations. It must be noted that wellbeing is a multifaceted phenomenon that can be assessed by measuring a wide array of subjective and objective constructs. Although we aimed to include a comprehensive list of pertinent nodes, failure to incorporate all relevant nodes may lead to spurious edges and to a misrepresentation of the network structure [26]. Finally, we chose to focus largely on composite scores for most of our place and community variables, due to the formative nature of the concepts being measured [34] and topological overlap of indicators [26]. Choosing to construct networks at this level of granularity can impact the network characteristics [30].

## Conclusion

The rich and layered analyses presented here provide important information for policymakers to address spatial disparities in wellbeing. Using network analyses to understand the complex connections between individual, community and place-based factors that correlate with wellbeing adds significant value to existing models. Our findings highlight the challenges in considering individual and place characteristics as truly separate domains. These findings can support certain evidence-based interventions based on a more sophisticated understanding of how they can affect change to population-level subjective wellbeing.

## Supplementary Information

**Additional file 1 Table S1**. Details of variables included in network model.

**Additional file 2 Methods S1**. Description of edge weight accuracy and centrality stability. **Methods S2**. Statement regarding licence of SWENWEBS. **Figure S1**. Results from edge weight accuracy and strength stability tests. **Figure S2**. Network visualisation including demographic factors as nodes. **Figure S3**. Strength values of full network including demographic covariates. **Table S2**. Nonzero-edges connected to overall wellbeing node. **Table S3**. Nonzero-edges connected to specific wellbeing nodes.

## Data Availability

The data that support the findings of this study are available from National Institute of Health Research Collaboration for Leadership in Applied Health Research and Care North West Coast (NIHR CLAHRC NWC) but restrictions apply to the availability of these data, which were used under license for the current study, and so are not publicly available. For further details contact the corresponding author or CLAHRC NWC.

## Abbreviations

SWEMWBS: Short Warwick–Edinburgh Mental Wellbeing Scale
HiAP: Health in All Policy
NIHR CLAHRC NWC: National Institute of Health Research Collaboration for Leadership in Applied Health Research and Care North West Coast
PMRF: Pairwise Markov Random Field
PCA: Principal components analysis
