# Supplementary material for: The individual, place, and wellbeing – a network analysis
Source: BMC Public Health. 2021 Sep 6;21:1621. doi: 10.1186/s12889-021-11553-7 (PMC8419673; doi:10.1186/s12889-021-11553-7)
Supplement: Supplementary file 2 — Additional file 2 Methods S1. Description of edge weight accuracy and centrality stability. Methods S2. Statement regarding licence of SWENWEBS. Figure S1. Results from edge weight accuracy and strength stability tests. Figure S2. Network visualisation including demographic factors as nodes. Figure S3. Strength values of full network including demographic covariates. Table S2. Nonzero-edges connected to overall wellbeing node. Table S3. Nonzero-edges connected to specific wellbeing nodes. [file 12889_2021_11553_MOESM2_ESM.docx]

**The individual, place, and wellbeing – a network analysis**

Supplementary materials

Contents

[Methods S1. Description of edge weight accuracy and centrality stability 2](#_Toc63702553)

[Methods S2. Statement regarding licence of SWENWEBS 3](#_Toc63702554)

[Figure S1. Results from edge weight accuracy and strength stability tests. 4](#_Toc63702555)

[Figure S2. Network visualisation including demographic factors as nodes. 5](#_Toc63702556)

[Figure S3. Strength values of full network including demographic covariates. 6](#_Toc63702557)

[Table S2. Nonzero-edges connected to overall wellbeing node 7](#_Toc63702558)

[Table S3. Nonzero-edges connected to specific wellbeing nodes 8](#_Toc63702559)

## Methods S1. Description of edge weight accuracy and centrality stability

Edge weight accuracy refers to the degree of confidence with which we can interpret the ranking of the edge weights (strongest to weakest). To assess the accuracy of the networks, bootstrapped 95% confidence intervals (CIs) were calculated for each edge, and the results plotted. A lack of overlap between confidence intervals indicates a significant difference in the strength of two edges (Epskamp, Borsboom, & Fried, 2018). Centrality stability refers to the reliability of the rank ordering of the centrality indices. This was examined using the case-dropping subset bootstrap method (Epskamp, Borsboom, & Fried, 2018); networks were re-estimated using increasingly smaller subsets of the original sample, and correlations between the original centrality indices and the subset centrality indices were calculated. A small-to-moderate decrease in correlation as participants are removed suggests that the order of centrality is relatively stable/reliable. This can be quantified in the form of the correlation stability coefficient, with values above 0.7 deemed to reflect high centrality reliability, and values between 0.25 and 0.7 denoting moderate reliability (Epskamp, Borsboom, & Fried, 2018). Accuracy and reliability analyses were conducted using the R package ‘bootnet’, based on 5,000 bootstrapped samples (Epskamp, Borsboom, & Fried, 2018).

## Methods S2. Statement regarding licence of SWENWEBS

The SWEMWBS is free to use if permission is sought from (https://warwick.ac.uk/fac/sci/med/research/platform/wemwbs/using), which was received by the NIHR CLAHRC NWC in 2015. SWEMWBS is protected by copyright:

“Short Warwick Edinburgh Mental Wellbeing Scale (SWEMWBS) © NHS Health Scotland, University of Warwick and University of Edinburgh, 2008, all rights reserved.”


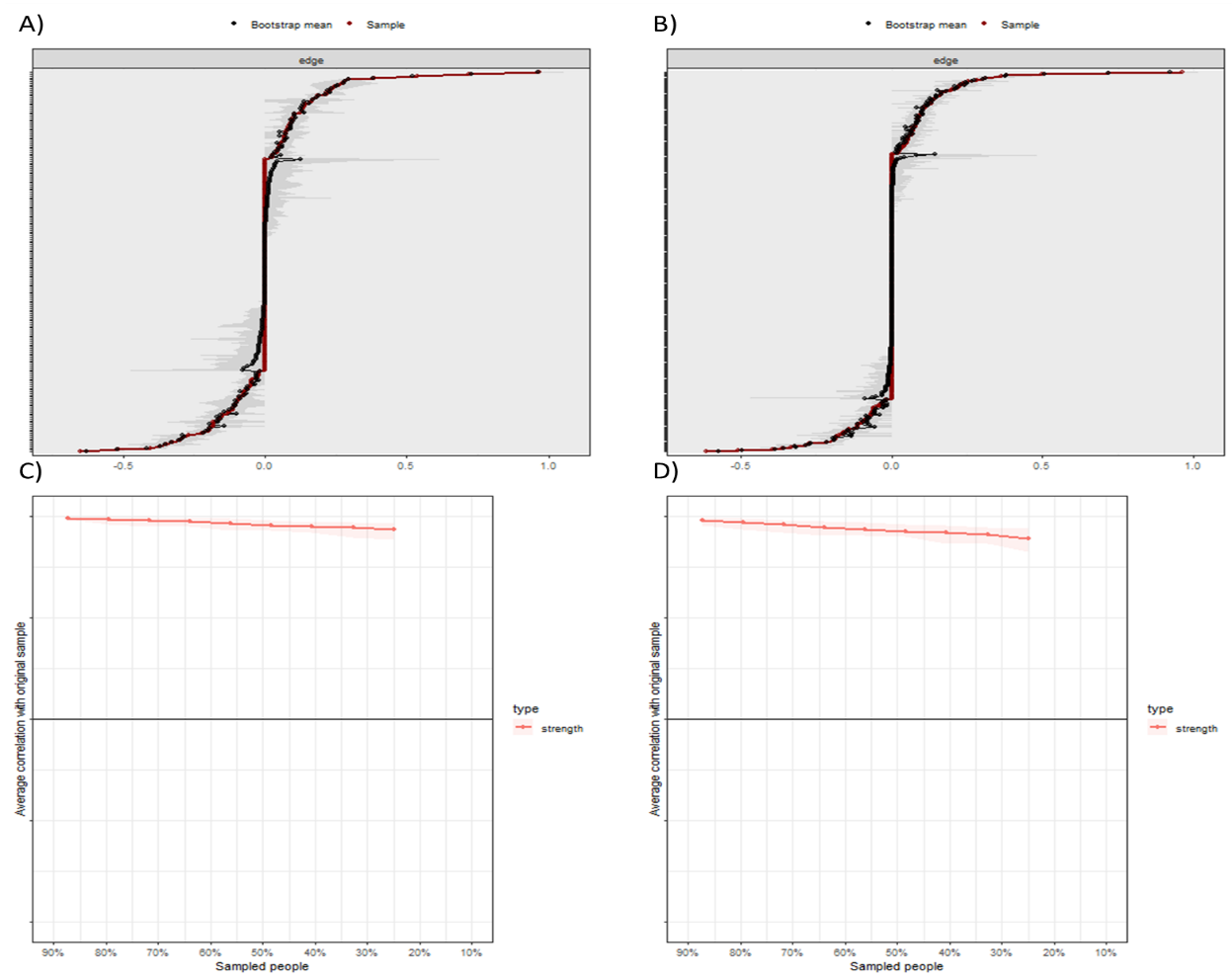


Figure S1. Results from edge weight accuracy and strength stability tests. Correlation stability coefficients all above recommended value of 0.7. Panel A = overlapping confidence intervals in network with overall wellbeing score node. Panel B = overlapping confidence intervals in network with individual SWEMWBS items. Panel C = correlation of centrality measures from random subsets with those of full sample (overall wellbeing node). Panel D = correlation of centrality measures from random subsets with those of full sample (individual SWEMWBS items).


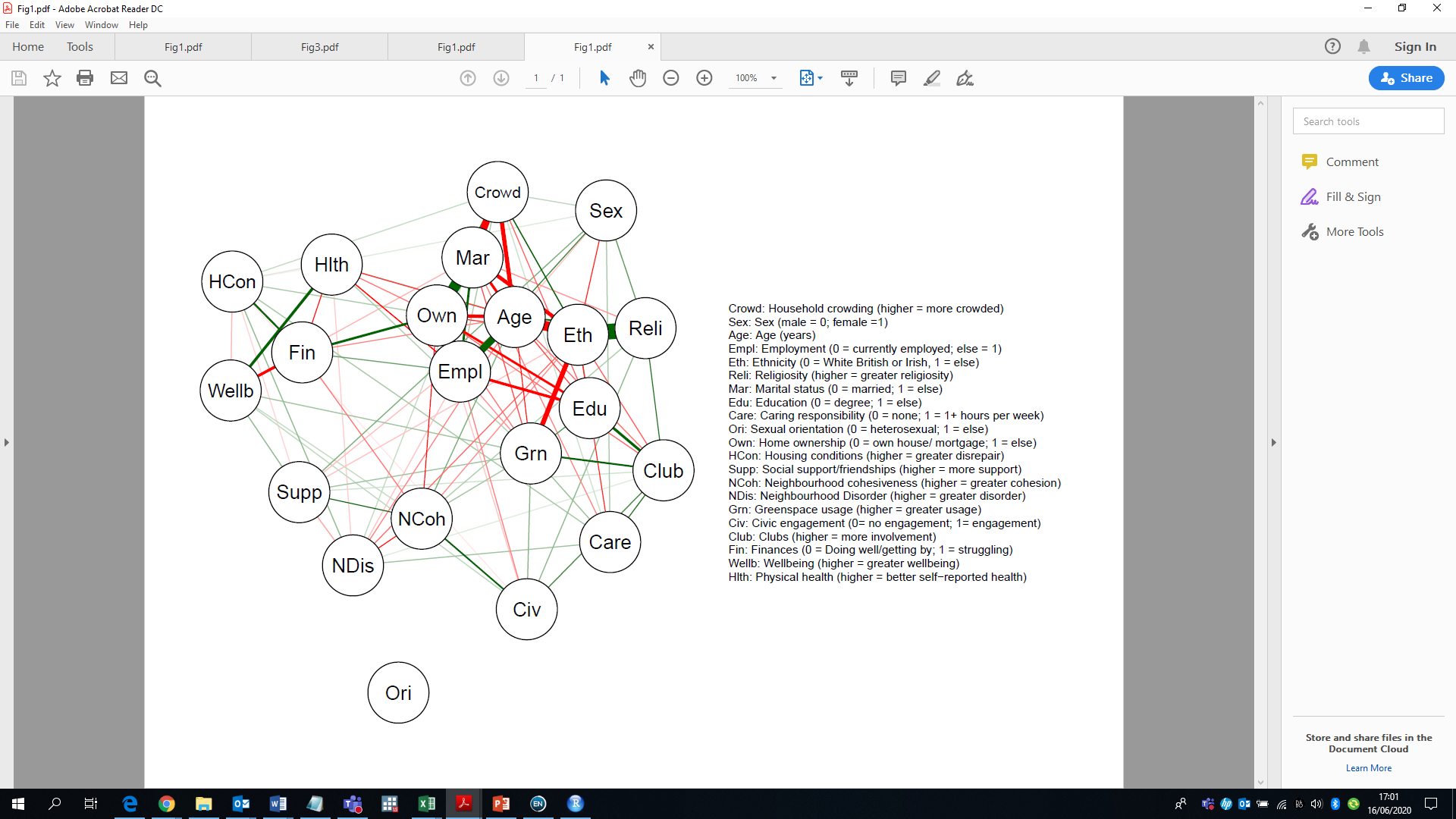


## Figure S2. Network visualisation including demographic factors as nodes.


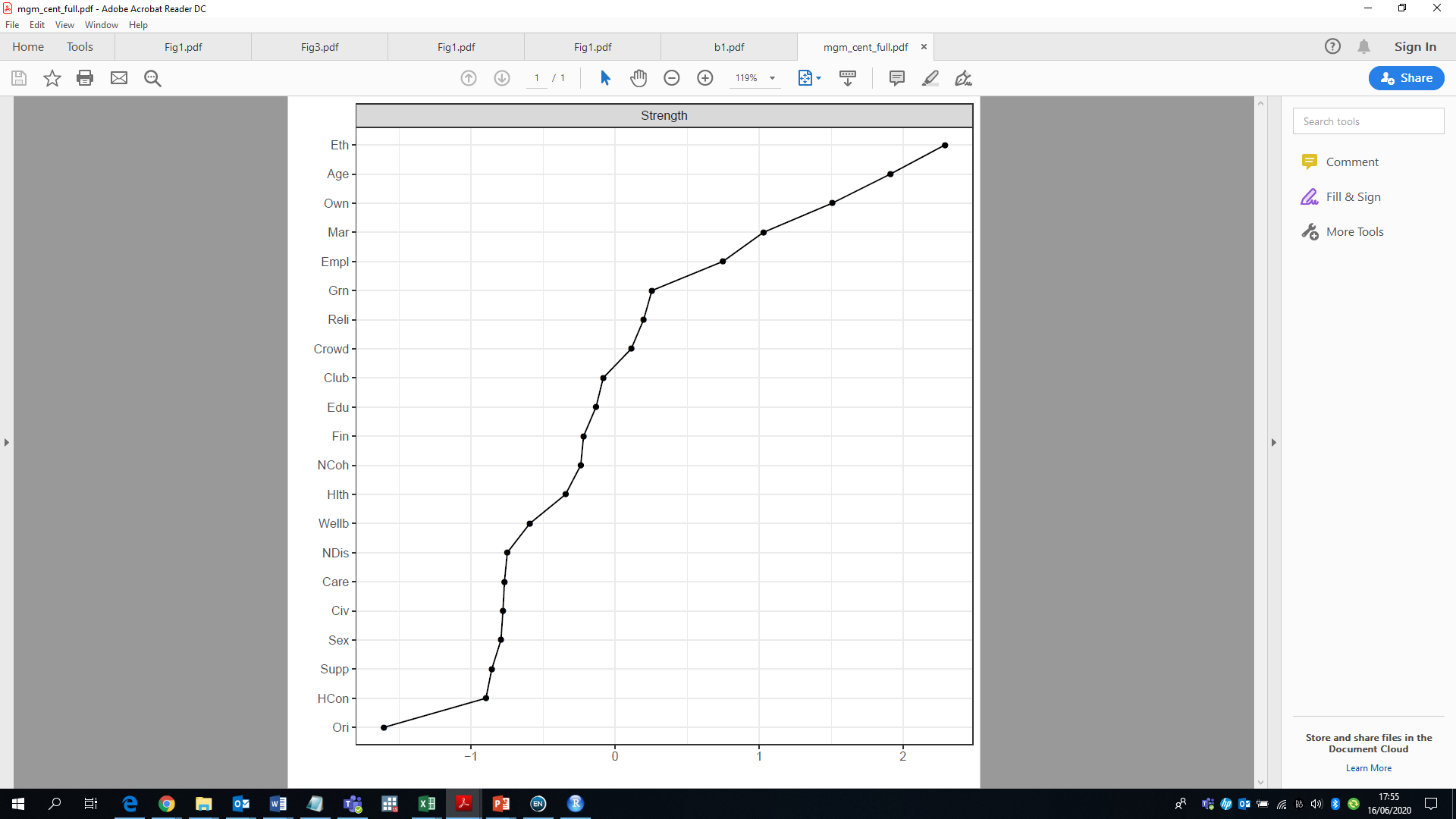


## Figure S3. Strength values of full network including demographic covariates.

| Table S2. Nonzero-edges connected to overall wellbeing node | |  |
| --- | --- | --- |
| **Node** | **Characteristic type** | **Weight** |
| Finances (0 = Doing well/getting by; 1 = struggling) | Individual | -0.30 |
| Subjective health (higher = better self-reported health) | Individual | 0.29 |
| Social support/friendships (higher = more support) | Individual | 0.09 |
| Greenspace usage (higher = more usage) | Place | 0.08 |
| Marital status (0 = married; 1 = else) | Individual | -0.06 |
| Housing condition (higher = greater disrepair) | Place | -0.06 |
| Civic agency (0= no engagement; 1= engagement) | Place | 0.05 |
| Neighbourhood cohesion (higher = greater cohesion) | Place | 0.05 |

*Note*. Edge weights are the arithmetic mean of regression coefficients between two nodes

(e.g mean of wellbeing → finance and finance → wellbeing)

| Table S3. Nonzero-edges connected to specific wellbeing nodes | | | | | | |
| --- | --- | --- | --- | --- | --- | --- |
|  | **Characteristic type** | **Optimistic** | **Useful** | **Relaxed** | **Dealing with problems** | **Closeness** |
| Employment (0 = currently employed; else = 1) | Individual | -0.03 | -0.06 | -0.07 | - | - |
| Religiosity (higher = greater religiosity) | Individual | 0.03 | - | - | - | - |
| Marital status (0 = married; 1 = else) | Individual |  | - | - | - | -0.08 |
| Education (0 = less than degree; 1 = degree) | Individual | 0.09 | - | - | - | - |
| Caring responsibility (0 = none; 1 = 1+ hours per week) | Individual | - | - | -0.05 | - | - |
| Social support/friendships (higher = more support) | Individual | - | - | - | - | 0.15 |
| Neighbourhood cohesiveness (higher = greater cohesion) | Place | - | - | - | - | 0.08 |
| Greenspace usage (higher = greater usage) | Place | - | 0.05 | - | - | 0.03 |
| Civic agency (0= no engagement; 1= engagement) | Place | 0.09 | - | - | - | - |
| Struggling financially (0 = Doing well/getting by; 1 = struggling) | Individual | - | - | -0.16 | -0.05 | - |
| Subjective health (higher = better self-reported health) | Individual | - | 0.11 | 0.09 | 0.04 | - |

*Note*. Edge weights are the arithmetic mean of regression coefficients between two nodes (e.g mean of employment → optimistic and optimistic → employment)
